# Photoactive cotton fabric for UV protection and self-cleaning

**DOI:** 10.1039/c9ra02023c

**Published:** 2019-06-10

**Authors:** Ishaq Ahmad, Chi-wai Kan, Zhongping Yao

**Affiliations:** Institute of Textiles and Clothing, The Hong Kong Polytechnic University Hung Hom Kowloon Hong Kong ahmadrai621@gmail.com tccwk@polyu.edu.hk; Department of Applied Biology and Chemical Technology, The Hong Kong Polytechnic University Hung Hom Kowloon Hong Kong zhongping.yao@polyu.edu.hk

## Abstract

Development of textile materials with tailored properties by coating with nano-materials is an emerging field of research. The preparation and characterization of photoactive cotton fabric for UV protection and self-cleaning properties are reported herein. In this study, a photoactive sol comprising of a reactive blue dye, C.I. Reactive Blue 21 (RB-21), and TiO_2_ was prepared by using sol gel method. The TiO_2_ nano sol was mixed with RB-21 to extend its photocatalytic activity in the visible region of the solar spectrum. The cotton fabric was coated with the RB-21/TiO_2_ sol *via* dip-pad-dry-cure method. Surface characterization of the coated cotton fabric was performed by FTIR-ATR, UV-visible absorption, XRD and SEM studies. FTIR-ATR and UV-visible spectra confirmed stable attachment of the photoactive RB-21/TiO_2_ coating on the cotton fabric. SEM images and XRD pattern shown the presence of anatase TiO_2_ on the coated cotton fabric. UV-protection, photocatalytic performance and self-cleaning properties of the coated cotton fabric were evaluated by the UV transmittance spectra, degradation of Rhodamine B (RhB) dye and stain removal under visible light respectively. Degradation of RhB was observed in the presence of RB-21/TiO_2_ coated cotton when exposed to visible light. Moreover, the coated cotton fabrics displayed excellent UV protection properties.

## Abstract 2

Development of textile materials with tailored properties by coating with nano-materials is an emerging field of research.

## Graphical abstract

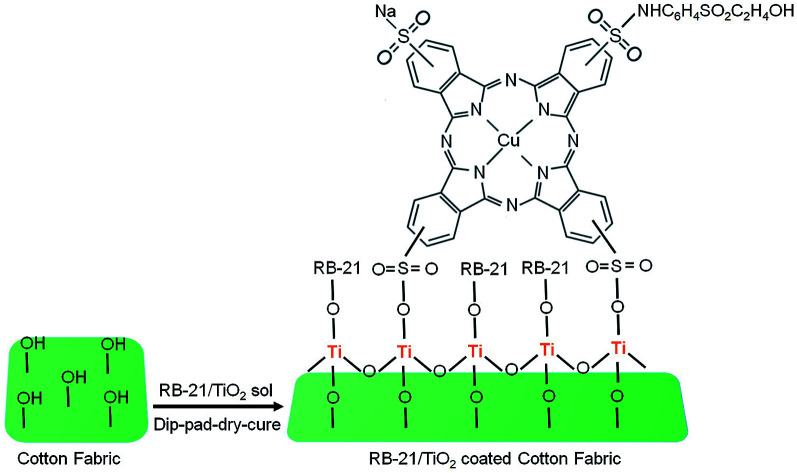

## 1 Introduction

Due to the extensive use of cotton fabrics in daily life, the integration of new functional properties in cotton fabrics has attracted particular attention. The inherent properties of cotton fabrics such as wettability, porosity, flexibility, absorbency, biodegradability and layered surface structure contribute to the integration of multiple functions such as anti-bacterial,^1^ self-cleaning,^2^ UV-blocking,^3^ bio-sensing,^4^ oil–water separation properties^5^ and smart textiles^6^ with many practical applications. Sol–gel, sputtering, sono-chemical, microwave-assisted, electrochemical, spraying, plasma treatment and laser vapor deposition coating methodologies have been used to incorporate functional materials on cotton fabric. Among these technologies, the sol–gel method is generally preferred due to its inexpensive and environmentally friendly productivity in coating technology.^7^ Metals, metal oxides, semiconducting metal oxides and polymers are primarily used to impart unique properties to cotton fabrics. Strong adhesive, photocatalytic, UV blocking and non-toxic properties of nanoscale titanium dioxide (TiO_2_) make it the most promising material applied on the cotton fabrics for tailored properties.^8,9^ Moreover, nanoscale TiO_2_ can be prepared by the cheaper and environmentally friendly methods. Recently, the integration of UV protection and self-cleaning properties in the textile materials by photoactive TiO_2_ coating has received extensive attention.^10,11^

The TiO_2_ coated fabric exhibits significant photocatalytic properties and degrades stains and dyes upon exposure to ultraviolet light. However, the TiO_2_ utilization as a catalyst in the development of commercial scale self-cleaning textiles has several limitations. The first limitation of TiO_2_ coating on cotton fabrics is its weak attachment to the fabric surface. The second limitation is its absorption of light only in the UV range of the solar spectrum, which accounts for only 4% to 5% of the entire spectrum. The first limitation of TiO_2_ coating on woven fabrics has been addressed by introducing some polar functional groups on the fabric surface *via* pretreatment processes. Chemical,^12^ radiofrequency plasma, microwave plasma,^13^ vacuum UV radiation and UV-C radiation^14^ pretreatments of the cotton fabrics impart COO^−^, –O–O^−^, lactams, phenols and other organic anions on the cotton fabric. These negatively charged active sites facilitate the stable attachment of TiO_2_ on fabric surface. To overcome second limitation of the TiO_2_ as a photocatalyst, many strategies are under study to make TiO_2_ visible light active. Doping the TiO_2_ with noble metals like silver (Ag)^15,16^ and gold (Au)^17,18^ and with non-metals like nitrogen^19^ has been reported to enhance its visible light photocatalytic efficiency of TiO_2_. In addition, the combined photocatalytic effect of TiO_2_ with other semiconductor metal oxides like SiO_2_ have also been studied for incorporation of self-cleaning properties in the cotton fabrics.^20^ However, the photocatalyst stability has been a challenging aspect for metal and not metal doped TiO_2_. Moreover, attachment of a photosensitizer, porphyrin (a chlorophyll analogue), on the surface of TiO_2_ has been reported which harvest the visible light of solar energy and increase the electron density in the conduction band of TiO_2_. This increased electron density enhances its photocatalytic activity.^21^ However, porphyrin synthesis and purification are very complicated and costly processes thus can't be used on industrial scale for practical applications. In this study, a reactive dye, C.I. Reactive Blue 21 (RB-21), has been selected for visible light harvesting. RB-21 is a phthalocyanine (PC) based dye with copper central metal atom in the PC ring. It has widely been used in the dyeing sector of textile industries. RB-21 was mixed in TiO_2_ nano-sol during sol gel method and its combined photocatalytic self-cleaning effect on cotton fabric has been reported herein.

## 2 Experimental

### 2.1 Materials

**Fig. 1:**
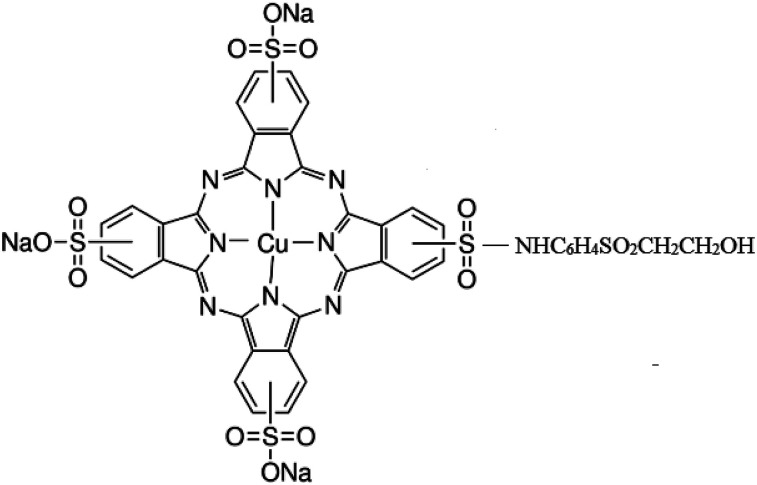
Chemical structure of Reactive Blue 21 (RB-21).

Titanium tetraisopropoxide (TTIP, Aldrich 97%), a TiO_2_ precursor was purchased from Sigma Aldrich and C.I. Reactive Blue 21 (89% dye content) was received from Avani Dye Chem Industries, India. The chemical structure of RB-21, as provided, is given in Fig. 1. Plain-woven cotton fabric having areal density of 119 g m^−2^ was selected for this study. Ne 40/1 100% cotton yarn was used in both warp and weft directions. The warp and weft densities of the cotton fabric were 52 cm^−1^ and 28 cm^−1^ respectively.

### 2.2 Preparation of RB-21/TiO_2_ sols

Three sets of TiO_2_ sols with composition of 5% TTIP, 70% H_2_O, 20% absolute ethanol, 4% glacial acetic acid and 1% hydrochloric acid were prepared separately. Titanium tetraisopropoxide (TTIP), a TiO_2_ precursor was dissolved separately in absolute ethanol and poured dropwise to the solvent medium. The mixture was heated at 70 °C for 16 h with constant stirring. RB-21 solution (0.05 g/100 mL) was prepared in distilled water. 3 mL, 5 mL and 7 mL of RB-21 solution were added separately in the TiO_2_ sols and mixed thoroughly before coating on the cotton fabric. The RB-21/TiO_2_ sols were coded as RB-21 (a) with 3 mL of RB-21 solution, RB-21 (b) with 5 mL of RB-21 solution and RB-21 (c) with 7 mL of RB-21 solution.

### 2.3 Coating of cotton fabric with RB-21/TiO_2_ nano-sol

To remove the impurities from pre-scoured and bleached cotton fabric, it was washed with a non-ionic detergent (1 g L^−1^) before coating process. The cotton fabric was dried at 80 °C for 30 min in a preheated oven. Three pieces of the known weight of cotton fabric (20 × 20 cm) were dipped separately in RB-21 (a), RB-21 (b) and RB-21 (c) sols for 5 min. One piece of the cotton fabric was dipped in pure TiO_2_ sol with no RB-21 content. For homogeneous coating, the cotton fabrics dipped in different photoactive sols were pressed in a padding machine (Rapid Labortex Co., Ltd., Taipei, Taiwan) with padder at a constant nip pressure of 2.5 kg cm^−2^. The padded cotton fabrics were weighed to determine the wet pick up which was about 75–77%.^8^ Na_2_CO_3_ aqueous solution was thoroughly sprayed by conventional spraying method to neutralize the fabric surface. The coated photoactive cotton fabrics were heated in a pre-heated oven at 80 °C for 5–8 min till complete drying. The dried fabrics were cured in a curing machine (Mathis Lab dryer Labor-Trockner Type LTE, Werner Mathis AG Co., Oberhasli, Switzerland) at 120 °C for 5 min.

**Fig. 2:**
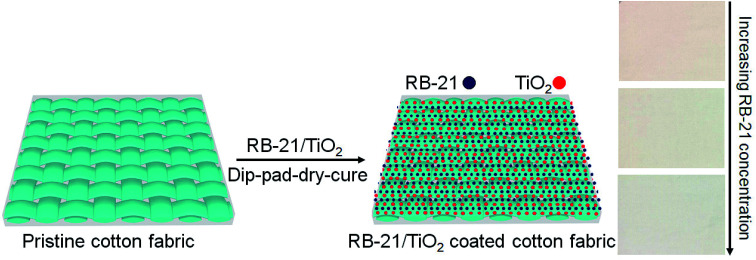
The schematic coating process of RB-21/TiO_2_ on the cotton fabric.

To remove the unattached dye molecules and TiO_2_ on the cotton fabrics, the coated cotton fabric was washed with hot and cold distilled water. Final obtained TiO_2_ and RB-21/TiO_2_ coated cotton fabrics were dried at 80 °C and saved at standard atmospheric conditions for characterization. The schematic coating process is given in the Fig. 2.

### 2.4 Fourier transform infrared (FTIR-ATR) spectroscopy analysis

Fourier transform infrared (FTIR-ATR) spectroscopy was used to elucidate the surface chemical characterization of the coated cotton fabrics. The FTIR-ATR transmittance spectra were recorded for both pristine cotton fabric and coated photoactive cotton fabrics. The specifications of lab FTIR-ATR spectrophotometer (Spectrum 100, PerkinElmer Ltd., Thane, India) were set at 16 cm^−1^ resolution with an average of 64 scans in the scanning range of 650–4000 cm^−1^ to get the transmittance spectra for each sample.

### 2.5 UV-visible absorption measurements

To study the absorption changes in the RB-21 dye molecules and TiO_2_, the UV-visible absorption spectra were recorded for RB-21 aqueous solution before coating and after coating on the cotton fabrics. The UV-visible absorption spectrum of RB-21 aqueous solution was recorded by a UV-visible UH5300 spectrophotometer (Hitachi, Tokyo, Japan) and the UV-visible absorption spectra of pristine cotton fabric, TiO_2_ coated and RB-21/TiO_2_ coated fabrics were recorded with Cary 300 spectrophotometer.

### 2.6 Color yield measurement

The RB-21 dye has inherent blue color. As we used very small concentration of RB-21 dye in the RB-21/TiO_2_ sols, after coating on the cotton fabric, there was no apparent blue color. Color yield measurements confirm the availability and amount of dye absorbed on the cotton fabrics. To measure the color yield of the RB-21 (a), RB-21 (b) and RB-21 (c) coated cotton fabrics, light reflectance on the fabrics surface was recorded by a reflectance spectrophotometer (Macbeth Color-Eye 7000A, Grand Rapids, Michigan) for each sample. The reflectance data was taken three times by using a D65 illuminant and 10° standard observer from 400 to 700 nm with 10 nm intervals. The *K*/*S* values gives information about the dye uptake by the cotton fabrics. The *K*/*S* values for all samples were calculated from reflectance data by using the Kubelka–Munk equation (eqn (1)). Lower the *K*/*S* value, lower is the dye uptake resulting in lower color yield and *vice versa*.1
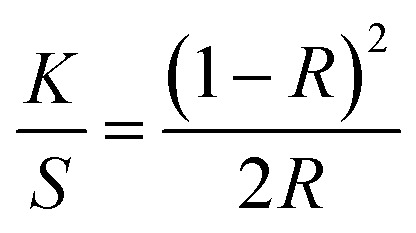
where *K* is the absorption coefficient of the RB-21 dye, *S* is the scattering coefficient and *R* is the reflectance of the coated cotton fabrics. The RB-21 (a), RB-21 (b) and RB-21 (c) coated cotton fabric were washed 5 times for 40 min at room temperature and exposed to light source for 30 h. The *K*/*S* values were measured after 5 washings and after light exposure to evaluate the laundering durability of the coated fabrics and photostability of the dye molecules respectively.

### 2.7 XRD and SEM analysis

TiO_2_ exhibits amorphous and three crystal structures. Three crystal structures are (I) anatase, (II) rutile and (III) brookite. To confirm the presence and determine the crystal structure of the TiO_2_ incorporated on the cotton fabric, XRD pattern of the pristine and the coated cotton fabrics were recorded by a high-power X-ray diffractometer (Rigaku Smartlab) while a Scanning Electron Microscope (Tescan VEGA3) was used to get SEM images of the pristine and the coated cotton fabrics to evaluate the surface morphologies.

### 2.8 Photocatalytic activity measurements

To evaluate the photocatalytic efficiency of the RB-21/TiO_2_ coated cotton fabrics, Rhodamine B (RhB) was used as a probe dye. RB-21/TiO_2_ coated cotton fabrics were used a photocatalyst for the photodegradation of RhB. Pristine cotton fabric, TiO_2_, RB-21 (a), RB-21 (b) and RB-21 (c) coated cotton fabrics (3 g each) were cut into small pieces. Standard aqueous solution (18 mg L^−1^) of RhB was prepared for the photocatalytic study. 100 mL of the standard solution was taken in five different 250 mL glass beakers. Pristine cotton fabric, TiO_2_, RB-21 (a), RB-21 (b) and RB-21 (c) coated cotton fabrics pieces were thoroughly dipped in the different RhB standard solutions. RhB solutions with pristine cotton and photoactive coated cotton fabrics were placed in dark for 2 h to get absorption–desorption equilibrium. Then, the beakers with test specimens were placed in a light box under a visible light source with constant stirring. Philip fluorescent lamps were used as visible light source and the light intensity on top of each test specimen was measured as 5.2–5.3 mW cm^−2^. 10 mL of the RhB solution from test samples was taken out after 30 min from each sample for 3 h and absorption spectra was recorded by using a UV-visible UH5300 spectrophotometer (Hitachi, Tokyo, Japan). Concentration of the probe dye after regular time intervals of 30 min was measured from absorption data and compared with the initial dye concentration. The photocatalytic performance of the photoactive cotton fabrics was evaluated by calculation the probe dye degradation efficiency by using eqn (2).
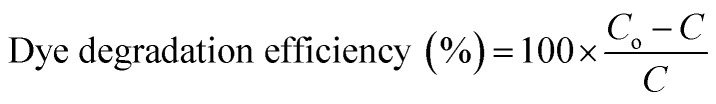
where *C* is concentration of RhB at any specific time and *C*_o_ is initial concentration of RhB. The relative decrease in the concentration of RhB with time under visible light source was examined by plotting *C*/*C*_o._

### 2.9 UV-protection factor analysis

To measure the UV protection factor, the UV transmittance spectra of pristine cotton, RB-21 (a), RB-21 (b) and RB-21 (c) coated cotton fabrics were recorded by Cary 300 spectrophotometer. The transmittance spectra were recorded twice for each sample at a right angle with the scanning speed of 300 nm min^−1^ at wavelength range of 280–400 nm. The UV-protection results were calculated by Cary 300 using the methods Australian/New Zealand Standard (AS/NZS 4399:1996) was used to evaluate the UV protection factor.

### 2.10 Self-cleaning studies

The photoactive cotton fabrics can also degrade the stains present on its surface in the presence of light. This characteristic of the coated cotton fabric is termed as self-cleaning property. The self-cleaning efficiency of the RB-21 (a), RB-21 (b) and RB-21 (c) coated cotton fabrics was evaluated by degradation of the stains. The stain solution of RhB with known concentration (7.5 mg L^−1^) was prepared and the coated cotton fabrics with dimensions of 5 × 2.5 cm were dipped in it. The stained fabric samples were taken out from the solution and dried in dark and then exposed to light (Philips TL-D 18 W fluorescent lamp with full used) for 3 h.

### 2.11 Washing test

The RB-21/TiO_2_ coated cotton fabrics were washed five times in a laundry machine for 40 min at room temperature in the absence of any detergent to check the washing stability the photoactive coating.

## 3 Results and discussion

### 3.1 Fourier transform infrared spectroscopy analysis

**Fig. 3:**
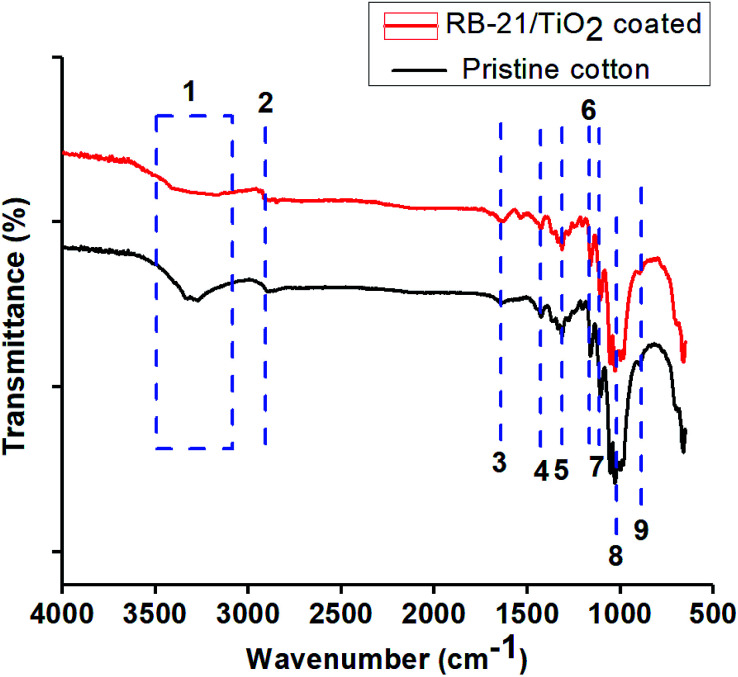
FTIR-ATR transmittance spectra of pristine cotton and RB-21/TiO_2_ coated cotton fabric.

Chemical surface modification of the cotton fabric by coating with RB-21/TiO_2_ was studied by FTIR analysis. The FTIR-ATR spectra were taken in transmittance mode for pristine and the coated cotton fabrics. The spectra are given in the Fig. 3. All FTIR-ATR characteristic peaks are given in the Table 1. From Fig. 3, it can be observed that the broad peak at 3500–3100 cm^−1^ in the spectra of RB-21/TiO_2_ coated cotton fabrics has reduced in peak intensity which indicates that the surface hydroxyl (OH) groups the cotton fabric has been occupied by the RB-21/TiO_2_ coating.^22^

**Table 1:** The FTIR-ATR transmittance characteristic peaks of cotton fabrics

Peak number	Wavenumber (cm^−1^)	Peak characteristics
1	3500–3100	O–H stretching vibration of H-bonded hydroxyl groups
2	2916	–CH_2_ asymmetric stretching of long alkyl chain
3	1645	Adsorbed H_2_O
4	1433	–CH in plane bending
5	1314	–CH wagging
6	1163	Asymmetric bridge C–O–C
7	1106	Asymmetric bridge C–O–C
8	1028	C–O stretch
9	896	Asymmetric stretching of C1–O–C4 of cellulose

**Fig. 4:**
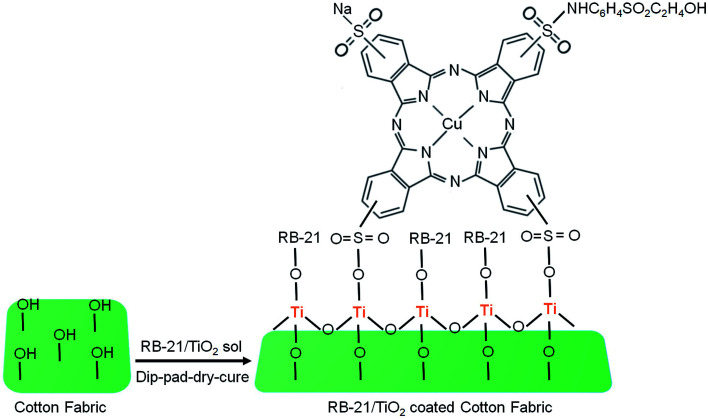
The structural schematic mechanism of the RB-21/TiO_2_ attachment on the cotton fabric surface.

Moreover, the decrease in peak intensity of stretching C–O at 1028 cm^−1^ in the FTIR spectra of coated fabric has been observed which also indicates the attachment of TiO_2_ on the fabric surface. The structural schematic diagram of the RB-21/TiO_2_ coated cotton fabric is given in Fig. 4.

### 3.2 UV-visible absorption analysis

**Fig. 5:**
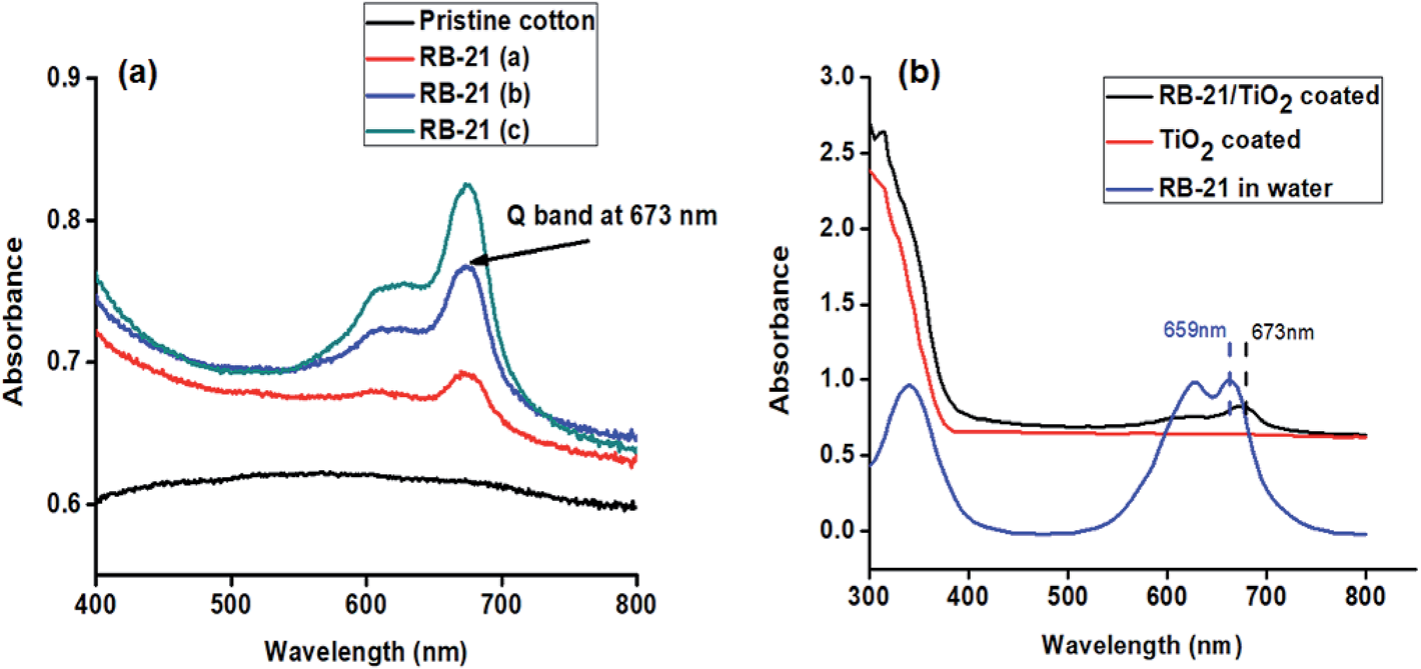
UV-visible absorption spectra of RB-21 aqueous solution and the cotton fabric coated with TiO_2_ and RB-21/TiO_2_.

Different dye concentrations of RB-21 were used in RB-21/TiO_2_ hybrids. The absorption spectra of pristine cotton and the cotton fabric coated with RB-21 (a), RB-21 (b) and RB-21 (c) are given in the Fig. 5(a). The absorption intensity increased with increasing dye concentration in the hybrid which was observed from the absorption spectra. The strong absorption peak, a characteristic peak of RB-21 (Q band) was observed at 673 nm. This absorption band can be observed in all three coated fabrics which confirms the presence of RB-21 in all three samples.

Attachment of RB-21/TiO_2_ on the fabric surface was also confirmed by the UV-visible absorption results. The UV-visible absorption spectra of RB-21 aqueous solution and the cotton fabrics coated with TiO_2_, RB-21/TiO_2_ are given in the Fig. 5(b). Maximum absorption (*λ* max) of RB-21 in water was observed at 659 nm. The maximum absorption (*λ* max) appeared at 673 nm and there was a red shift (bathochromic shift) of 14 nm after coating on the cotton fabric which indicates the stable anchoring of RB-21/TiO_2_ molecules on the fabric surface.^23^ Moreover, the presence of TiO_2_ on the fabric surface is also indicated in the absorption spectrum of RB-21/TiO_2_ coated cotton fabrics. The onset of absorption of TiO_2_ also shows a red shift towards higher wavelength when TiO_2_ was mixed with RB-21 and coated on the fabric which indicates the better visible light absorption and higher photocatalytic efficiency of the coated fabrics.

### 3.3 Color yield results

To evaluate the existence and durability of color on the coated fabrics due to the RB-21, the color yield measurements were conducted in three rounds; (I) after the coating of RB-21/TiO_2_ on to the cotton fabric, (II) after five washing cycles and (III) after 30 h exposure to a visible light source. The K/S values for all three rounds are given in the Table 2.

**Table 2:** The *K*/*S* values of the coated cotton fabrics with RB-21/TiO_2_

Cotton fabric specimen	*K*/*S*		
	After coating	After 5 washings	After 30 h light irradiation
RB-21 (a)	1.207	1.201	1.041
RB-21 (b)	2.562	2.547	1.461
RB-21 (c)	2.883	2.772	1.239

The *K*/*S* values show that the coated fabric has stable laundering durability. There is negligible change in the *K*/*S* values after five washings. However, about 57% and 43% decrease in the *K*/*S* values of the cotton fabrics coated with RB-21 (c) and RB-21 (b) respectively when exposed to light source for long time (30 h). This decrease in the *K*/*S* values can be ascribed to the self-degradation of RB-21 when exposed to a light source for long time.

### 3.4 X-ray diffraction studies

**Fig. 6:**
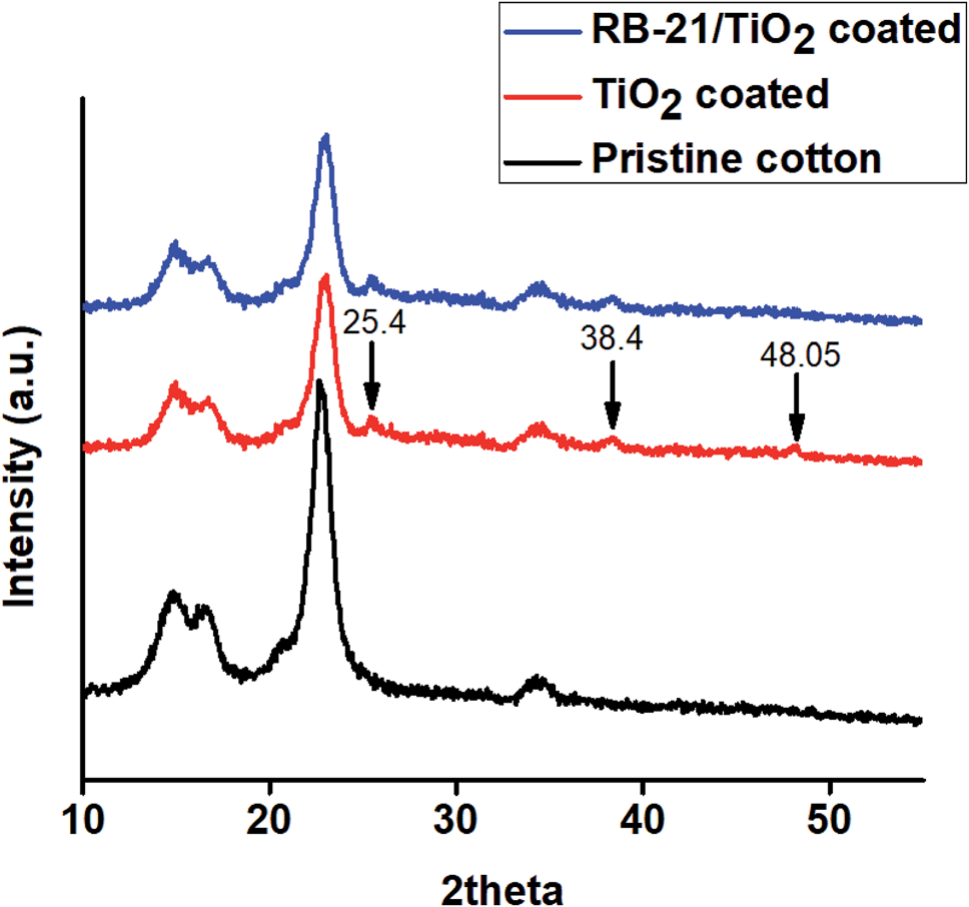
XRD pattern of pristine cotton, TiO_2_ coated and RB-21/TiO_2_ coated cotton fabric.

**Fig. 7:**
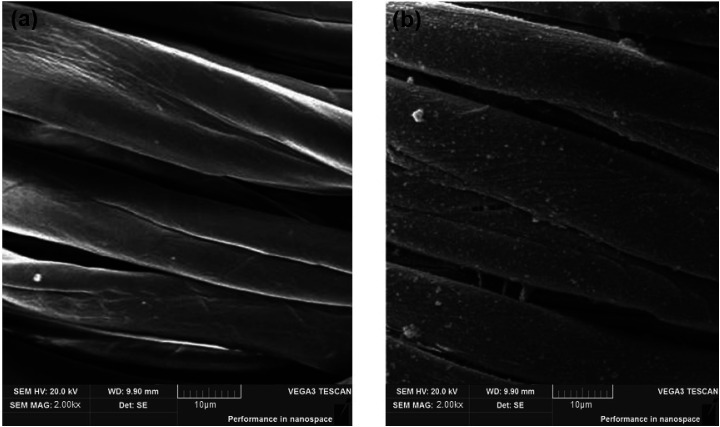
SEM images of (a) pristine cotton and (b) RB-21/TiO_2_ coated cotton fabric.

To observe the crystalline surface of the coated cotton fabrics, X-ray diffraction (XRD) studies were conducted. The obtained XRD graphs of pristine cotton and the cotton fabrics coated with TiO_2_ and RB-21/TiO_2_ are given in the Fig. 7. As pristine cotton is composed of polymeric cellulose chains which are arranged in specific crystalline phase. This cellulose crystalline structure give rise to sharp diffraction peaks in the XRD patterns which appear at 14.7°, 16.4°, 22.6° and 34.4° as shown in the Fig. 6.^24^

Three more diffraction peaks appeared in the XRD pattern at 25.4°, 38.4° and 48.05° after coating the cotton fabric with TiO_2_. These three diffraction peaks represent the anatase phase of TiO_2_ present on the fabric surface.^25^ The presence of characteristic peaks corresponding to the cellulose crystalline in the XRD pattern of TiO_2_ coated fabrics indicates that the coating of TiO_2_ on the cotton fabric surface does not affect the inherent crystalline structure of the cotton. Moreover, it was also observed from the XRD studies that anatase phase of TiO_2_ is retained after mixing with RB-21 as shown in the XRD pattern of RB-21/TiO_2_ coated cotton fabrics. The anatase phase of TiO_2_ is the most photoactive crystal structure and has more photocatalytic efficiency.

### 3.5 Scanning electron microscope (SEM) analysis

Scanning electron microscopic (SEM) studies of the coated cotton fabrics confirmed the deposition of photoactive material on the fabric surface. The deposition of the RB-21/TiO_2_ coating on the surface of cotton fabric was confirm by the of the coated cotton fabrics. SEM images of pristine and the RB-21/TiO_2_ coated cotton fabric are given in the Fig. 7.

**Fig. 8:**
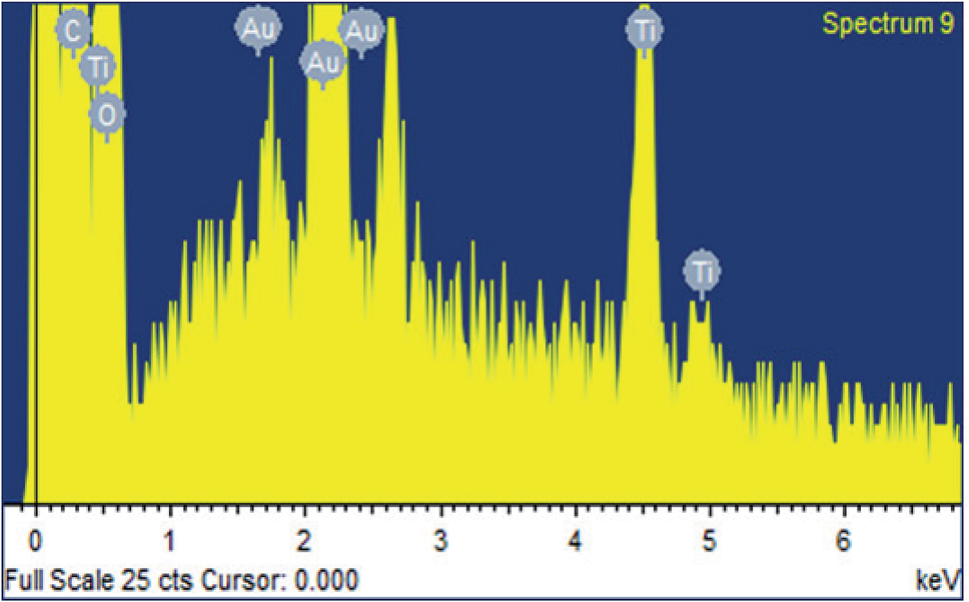
EDX spectrum of the cotton fabric coated with RB-21/TiO_2_.

The surface morphology of the pristine cotton fabric is shown in the Fig. 7(a). It has been observed that fabric surface is very smooth. However, the surface morphology changed when it was coated with RB-21/TiO_2_. The appearance of roughness as shown in the Fig. 7(b) on the coated fabric surface corresponds to the deposition of the photoactive RB-21/TiO_2_ on the fabric. In addition, the presence of TiO_2_ on the coated fabric surface was also confirmed from EDX data. The elemental EDX data collected for the coated cotton fabric has been given in the Table 3 and the EDX graph is given in the Fig. 8.

**Table 3:** The EDX data of the cotton fabric coated with RB-21/TiO_2_

Element	Weight percentage (%)	Atomic percentage (%)
Carbon (C)	41.27	53.37
Oxygen (O)	46.27	44.91
Titanium (Ti)	2.98	0.97
Gold (Au)	9.48	0.75

### 3.6 Photocatalytic studies

**Fig. 9:**
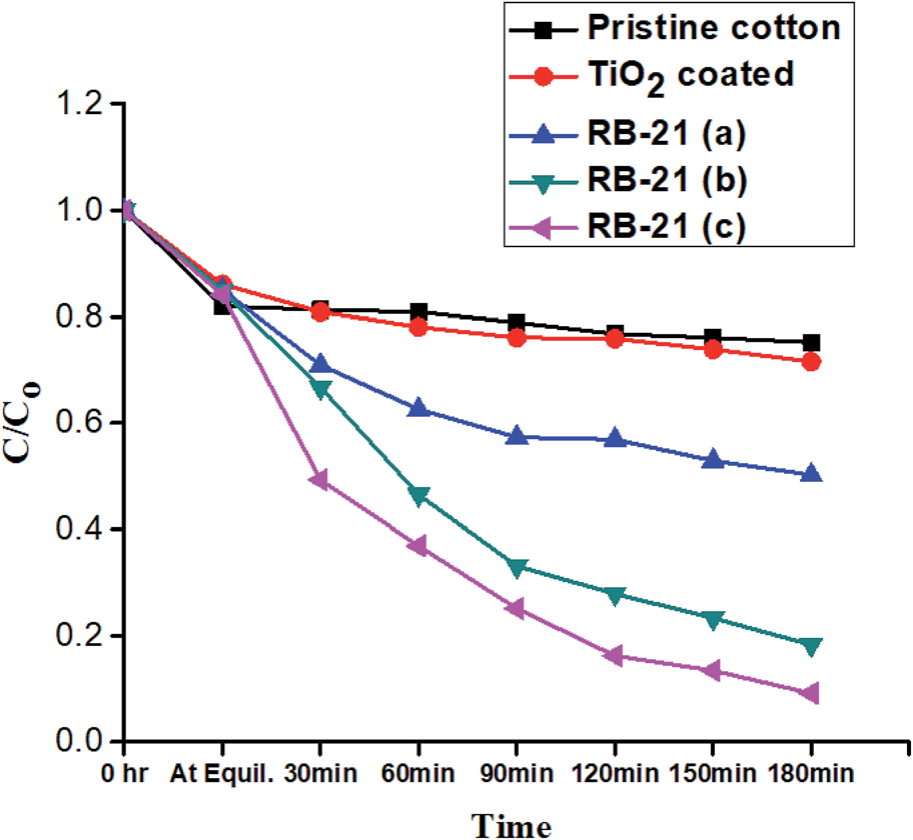
Photocatalytic degradation of the RhB.

Photocatalytic performance of the coated cotton fabrics was evaluated by the degradation of RhB in the presence of TiO_2_ coated and RB-21/TiO_2_ coated fabrics as a visible light source. The *C*/*C*_o_ values were drawn against time of light exposure and termed as degradation curves where *C*_o_ is initial concentration and *C* is the concentration of probe dye, RhB, at specific time of light exposure. All RhB degradation curves for pristine cotton, TiO_2_ coated and RB-21/TiO_2_ coated cotton fabrics are given in Fig. 9. No significant decrease in the RhB concentration was observed in the presence of pristine cotton and TiO_2_ coated cotton because pristine cotton and TiO_2_ are photo-inactive under visible light. However, RB-21/TiO_2_ coated cotton fabrics shown significant photocatalytic performance and significant decrease in RhB concentration was observed as shown in the degradation curves.

The photocatalytic performance of the final coated fabric was dependent on the concentration of the RB-21 dye. The degradation curves show a different photocatalytic efficiency for all three RB-21 (a), RB-21 (b) and RB-21 (c) coated cotton fabrics. RB-21/TiO_2_ coated cotton fabric with higher dye concentration shows higher photocatalytic efficiency as shown in the Fig. 9. This can be attributed to the excitation of greater number of RB-21 molecules by absorbing visible light and thus inject more electrons to the conduction band of TiO_2_. The higher electron density in the conduction band of the TiO_2_ results greater photocatalytic efficiency.

**Fig. 10:**
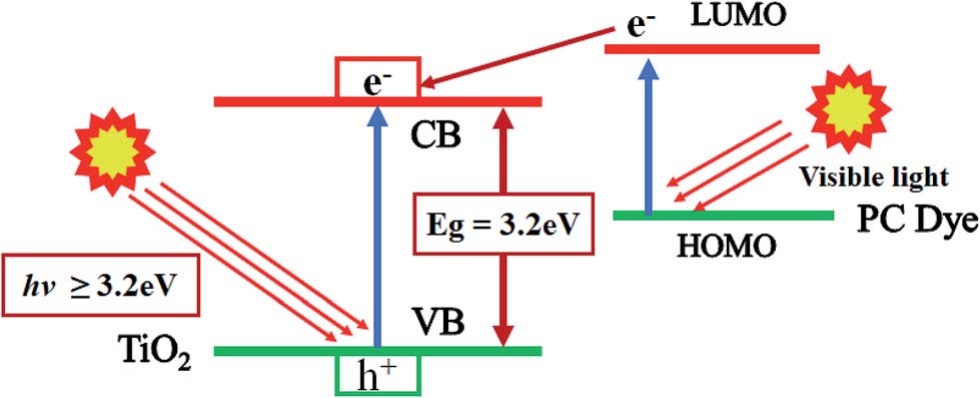
Schematic mechanism of photocatalytic mechanism of action of RB-21/TiO_2_.

The possible photocatalytic mechanism of action of RB-21/TiO_2_ can explained as follows. When RB-21/TiO_2_ coated cotton fabric is exposed to light source, the RB-21 molecules absorb visible light energy and electrons from highest occupied molecular orbital (HOMO) of RB-21 get excited to the lowest unoccupied molecular orbital (LUMO). The energy level of LUMO of RB-21 is higher than that of conduction band (CB) of the TiO_2_, thus these electrons are injected from LUMO to the CB of TiO_2_. The increased density in CB results in the increase in the photocatalytic efficiency of TiO_2_ under visible light source. The schematic photocatalytic mechanism is given in the Fig. 10.

### 3.7 UV protection results

The solar spectrum contains 3–5% of UV radiations which are hazardous to human health. The pristine cotton fabric is unable to absorb UV radiations which directly affect the human skin. To protect the human body from UV radiations, some UV absorbing clothing is necessary for outdoor activities. The photoactive cotton fabric developed in this study can absorb UV radiations and protect the body. UV protection factor, the potential of the fabric to absorb UV radiations, is used to evaluate the UV absorption efficiency of the fabric. UV protection factor (UPF) is a ranking of protective capabilities of textile fabrics against sun UV radiations. A UPF rating has been classified into 4 major parts according to the Australian/New Zealand Standard (AS/NZS 4399:1996). The textile fabric with UPF value less than 15 is ranked as non-ratable and has poor UV-blocking ability and is not suitable for outdoor wearing in sunlight exposure. The textile fabric with UPF value ranging from 15 to 50 is ranked as good to very good for UV-blocking. The textiles with UPF value of more than 50 are normally referred as excellent UV-blocking textiles.^17^ The UPF values of RB-21/TiO_2_ coated fabrics are given in the Table 4.

**Table 4:** UPF and UV light transmission (%) values for the cotton fabric coated with RB-21/TiO_2_

Sample name	UPF value	UV-A (%)	UV-B (%)	Status
Pristine cotton	6.9	31.4	29.7	
RB-21 (a)	150.076	6.571	0.194	1st Measurement
RB-21 (a)	136.029	7.157	0.233	After 5 washings
RB-21 (b)	162.841	5.854	0.210	1st Measurement
RB-21 (b)	157.833	5.982	0.241	After 5 washings
RB-21 (c)	127.227	7.395	0.241	1st Measurement
RB-21 (c)	112.631	7.581	0.325	After 5 washings

**Fig. 11:**
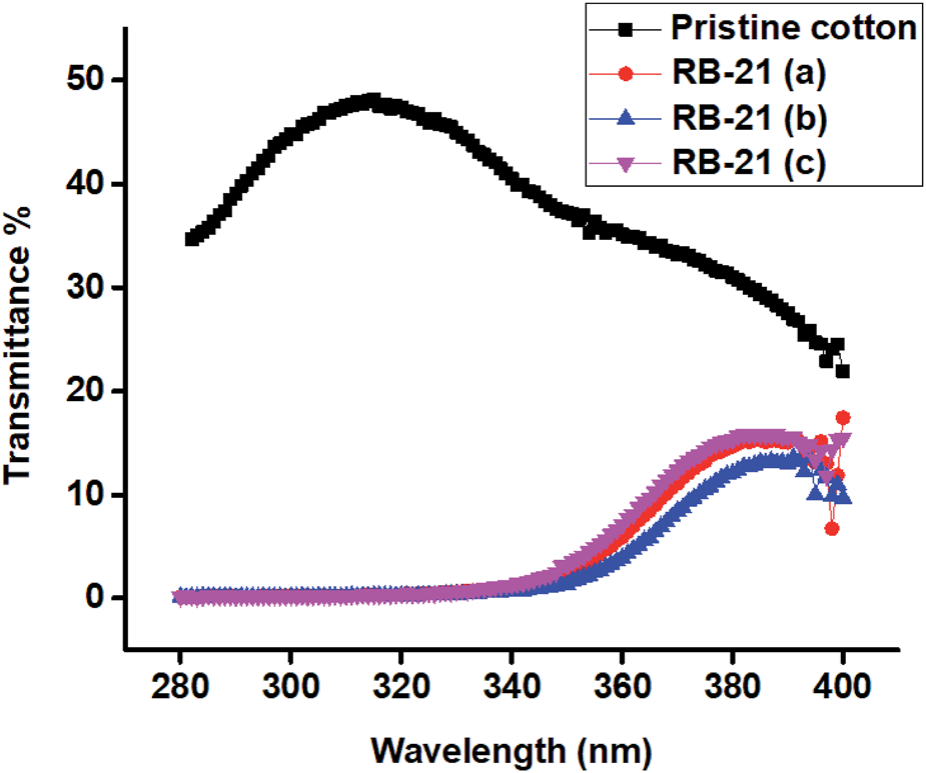
UV transmittance spectra of the cotton fabric coated with RB-21/TiO_2_.

It can be observed from the UPF and UV light transmission (%) values given in the table that the cotton fabrics coated with RB-21/TiO_2_ have excellent UV-protective properties. The UPF values for all RB-21/TiO_2_ coated fabrics are more than 100 which makes these fabrics excellent for wearing while engaging in outdoor activities under sunlight exposure. UV transmittance spectra of the coated fabrics given in the Fig. 11 also shows that they absorb almost all UV region of solar spectrum. There is negligible transmission of UV radiations.

In addition, this UV protective coating possesses high laundering durability. It can be observed from the UPF and UV light transmission (%) values given in the table that after five washings, each washing for 40 min at room temperature, RB-21/TiO_2_ coated fabrics retain their excellent UV-protective properties.

### 3.8 Self-cleaning studies

**Fig. 12:**
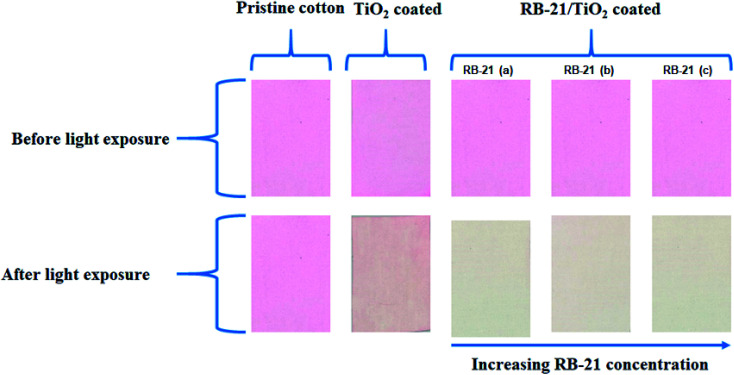
Self-cleaning results of pristine cotton, TiO_2_, RB-21 (a), RB-21 (b) and RB-21 (c) coated cotton fabrics.

For self-cleaning efficiency, pristine cotton, TiO_2_, RB-21 (a), RB-21 (b) and RB-21 (c) coated cotton fabrics stained with RhB dye were placed under light source. There was no effect of light on the stains present on the pristine cotton while little degradation of stains present on the TiO_2_ coated fabrics was observed. However, the cotton fabrics coated with RB-21 (a), RB-21 (b) and RB-21 (c) shown excellent self-cleaning results and almost all stains were removed from the surface as shown in the Fig. 12.

## 4 Conclusion and future work

In this study, preparation and characterization of photoactive cotton fabrics for UV protection and photocatalytic self-cleaning have been presented. Sol–gel method was used for the preparation of photoactive materials. The photoactive materials used in this study was based on a reactive dye, C.I. Reactive Blue 21 and TiO_2_. The photoactive TiO_2_ and RB-21/TiO_2_ sols prepared by the sol–gel method were coated on the cotton fabrics *via* dip-pad-dry-cure method. The wet pick up of photoactive TiO_2_ and RB-21/TiO_2_ by the cotton fabric was about 77%. Stable coating of TiO_2_ and RB-21 molecules on the cotton fabric was confirmed by FTIR, UV-visible and surface studies while photocatalytic self-cleaning efficiency was evaluated by the degradation a toxic dye, Rhodamine B (RhB) in the presence of the photoactive cotton fabric as a photocatalyst. The final coated cotton fabrics exhibited excellent UV protection and photocatalytic self-cleaning performance. However, the RB-21/TiO_2_ coated cotton fabrics showed some level of self-degradation of RB-21 when exposed to sun light for long time. From this limitation of the study, it may be suggested that more photostable phthalocyanine based reactive dyes can be prepared and used for self-cleaning cotton fabrics in future.

## Conflicts of interest

There are no conflicts to declare.

## Supplementary Material
